# Effect of Material Change on Stirnol Engine: A Combination of NiTiNOL (Shape Memory Alloy) and Gamma Stirling Engine

**DOI:** 10.3390/ma16083257

**Published:** 2023-04-20

**Authors:** Humayun Arif, Aqueel Shah, Tahir Abdul Hussain Ratlamwala, Khurram Kamal, Maqsood Ahmed Khan

**Affiliations:** 1 Department of I&ME, PNEC Pakistan Navy Engineering College, NUST, Karachi 75350, Pakistan; aqueel@pnec.nust.edu.pk (A.S.); tahir.ratlamwala@pnec.nust.edu.pk (T.A.H.R.); khurram.kamal@pnec.nust.edu.pk (K.K.); 2 Department of I&ME, NED University of Engineering and Technology, Karachi 75350, Pakistan; maqsoodahmed@neduet.edu.pk

**Keywords:** Shape Memory Alloy (SMA), Stirling engine, Stirnol engine, NiTiNOL, Stirling cryocooler, superelastic, Shape Memory Effect (SME), adiabatic chamber, Proportional Integral Derivative (PID) controller

## Abstract

Population explosion, industrialization, and urbanization have accelerated energy requirements across the globe. This has led to the human quest to find simple and cost-effective energy solutions. A promising solution is the revival of the Stirling engine with the addition of Shape Memory Alloy NiTiNOL in it. The experimental results reveal that the addition of a NiTiNOL spring at the base plate of the Stirling engine enhances the overall efficiency of the engine, demonstrating some impact of the shape memory alloy toward the performance output of the Stirling engine. The newly modified engine has been named the STIRNOL ENGINE. The comparative study of Stirling and Stirnol engines reveals a minuscule efficiency improvement, yet there is a furtherance that opens a window for future researchers to get a lead and venture into this new field. We are confident that with more complex designs and better Stirling and NiTiNOL combinations, more efficient engines can be invented in the future. This research focuses on changing the material of the base plate of the Stirnol engine and ascertaining its performance differential through the integration of the NiTiNOL spring. A minimum of four types of materials are utilized for experimentation.

## 1. Introduction

An important area of thermodynamics that has been utilized for a long time is power generation, which is usually governed under systems known as thermodynamic cycles. Some of the most commonly used power cycles are the Otto cycle, Carnot cycle, Diesel cycle, Stirling cycle, Brayton cycle, and jet propulsion cycles that govern the working principles of engines. These engines can be further categorized as internal combustion or external combustion engines. In external combustion engines (such as steam power plants), heat is supplied to the working fluid from an external source such as a furnace, a geothermal well, a nuclear reactor, or even the Sun. In internal combustion engines (such as automobile engines), this is performed by burning the fuel within the system boundaries. The heat engine discussed in this paper is a Stirling engine that functions on the principles of the Stirling cycle [1]. Said engine is an external combustion engine.

Shape memory alloys (SMAs) are one of the most widely used smart materials. These materials have the unique ability to remember their original shape under external load (superelastic) or thermomechanical load (shape memory effect) [2]. Owing to this unique characteristic, these materials are being used in many scientific types of research and a variety of industrial applications such as biomedical devices, automation, and robotics industries [3]. SMA elements can be of a variety of forms (strips, rods, sheets, wires, springs, tubes) in various sizes [4]. In the research that follows, spring-shaped NiTiNOL is used. NiTiNOL (Nickel Titanium alloy) was chosen for this experiment as the majority of SMAs are one-way while NiTiNOL is a two-way SMA. Moreover, such an SMA was to be chosen that was compatible with the internal temperature changes of the Stirling engine. Martensitic finish (M_f_) and Austenitic finish (A_f_) values of NiTiNOL are 30 °C and 60 °C, while the temperature range inside the Stirling engine is also maintained at 40–60 °C. In this regard, the best-suited SMA to be selected was NiTiNOL.

### 1.1. Stirling Engine Applications

**Figure 1:**
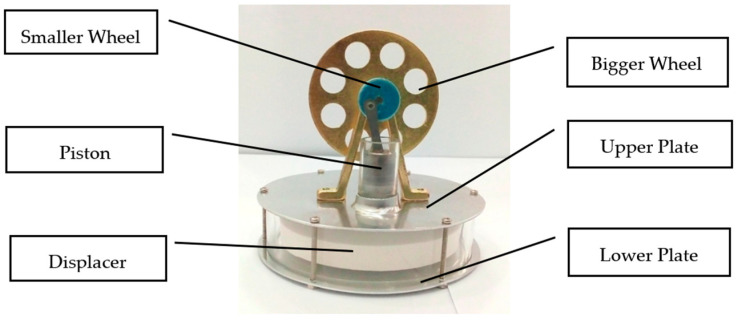
Low-Temperature Differential Gamma Stirling engine.

Stirling engines are used in multiple applications such as solar power generation [5,6], computer chip cooling [7], submarines [8], the domestic use of heat and power [9], vehicles [10,11], and the Stirling cryocooler [12]. Said engine comes in a variety of types and shapes; however, its basic shape known as a Low-Temperature Differential (LTD) gamma type Stirling engine is used in the research (refer to Figure 1).

### 1.2. The Stirling Cycle

**Figure 2:**
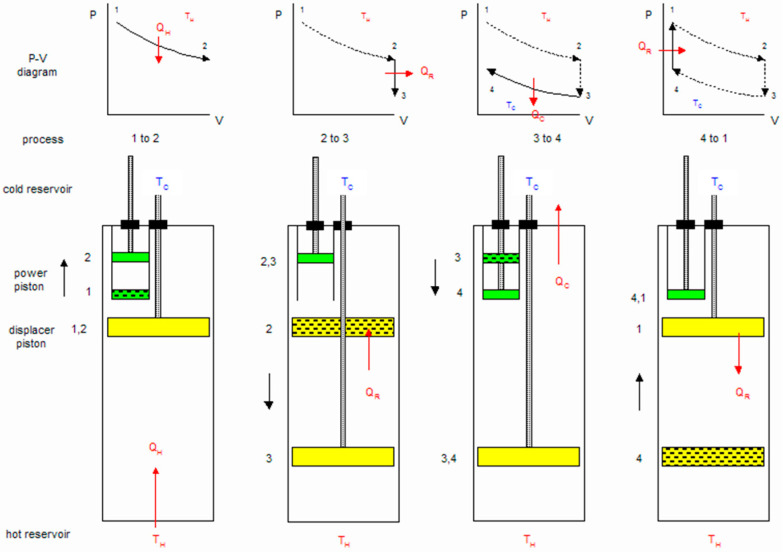
The Stirling Cycle.

The ideal Stirling cycle consists of four distinct thermodynamic processes as shown in Figure 2. A brief of all four processes is given in the subsequent paragraphs.

#### 1.2.1. Isothermal Expansion (1 to 2)

The expansion space and associated displacer are maintained at a constant high temperature, and the gas undergoes near-isothermal expansion by absorbing heat from the hot source.

#### 1.2.2. Constant-Volume (Known as Isovolumetric or Isochoric) Heat Removal (2 to 3)

The gas is passed through the displacer, where it cools, transferring heat to the displacer for use in the next cycle.

#### 1.2.3. Isothermal Compression (3 to 4)

The compression space and associated displacer are maintained at a constant low temperature, so the gas undergoes near-isothermal compression by rejecting heat to the cold sink.

#### 1.2.4. Constant-Volume (Known as Isovolumetric or Isochoric) Heat Addition (4 to 1)

The gas passes back through the displacer where it recovers much of the heat transfer in process 2, heating up on its way to the expansion space.

### 1.3. Power Efficiencies of Stirling Engines

The greatest drawback of Stirling engines is that they cannot achieve total efficiencies as compared to internal combustion engines with the main constraint being thermal efficiency. In internal combustion engines, temperatures rise to around 1500–1600 °C for a short period, resulting in a greater mean heat supply temperature of the thermodynamic cycle than any Stirling engine could achieve. Stirling engines operate at a lower temperature in the range of around 30–150 °C and can use almost any heat source.

### 1.4. Shape Memory Alloys (SMAs)

The breakthrough for engineering applications occurred with the discovery of Nickel Titanium alloy by Buehler and coworkers while investigating materials useful for heat shielding. It was found that in addition to its good mechanical properties, comparable to some common engineering metals, the material also possessed a shape recovery capability. The discovery of NiTiNOL led to active research interest in SMAs.

### 1.5. SMA Properties

Considering the typical operating temperature range, SMAs have two phases, each with a different crystal structure and different properties. One is the high-temperature phase called Austenite (A) and the other is the low-temperature phase called Martensite (M). Austenite (generally cubic) has a different crystal structure from martensite (tetragonal, orthorhombic, or monoclinic). The transformation from one structure phase to the other does not occur by the diffusion of atoms, but rather by shear lattice distortion. Such a transformation is known as martensitic transformation. The reversible phase transformation from austenite (parent phase) to martensite (product phase) and vice versa forms the basis for the unique behavior of SMAs.

### 1.6. Stirnol Engine

The modified Stirling engine by the addition of a NiTiNOL spring is termed the Stirnol engine.

## 2. Modification from Stirling Engine to Stirnol Engine

**Figure 3:**
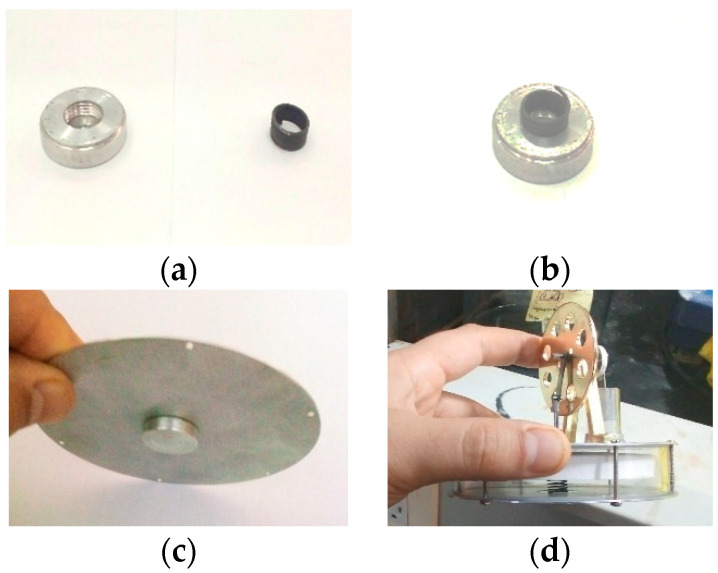
Stirnol engine with modified NiTiNOL Spring: (**a**) Cup-shaped threaded Aluminum holder with NiTiNOL spring; (**b**) Integration of cup-shaped threaded Aluminum holder with NiTiNOL spring; (**c**) Integration of cup-shaped threaded Aluminum holder and NiTiNOL spring with Stirling engines base plate; (**d**) Stirnol engine with NiTiNOL spring attached to its lower plate.

As stipulated above, the modification that differentiates the Stirling engine from the Stirnol engine is the addition of a NiTiNOL spring in it. The modification is actuated by manufacturing a cup-shaped threaded aluminum holder. This holder is threaded from inside to fix the spring in it. This cup-shaped aluminum holder is further assembled inside the lower plate in a push-tight arrangement to prevent air leakage, which might reduce the efficiency of the Stirnol engine. The complete arrangement of the Stirnol engine is shown in Figure 3, with its dimensions depicted in Table 1. To operate the engine, its lower plate is heated, which expands the spring and pushes the displacer up. Once the cool air touches the spring owing to the Stirling cycle, the spring returns to its original shape and contracts.

The NiTiNOL spring being used operates at low-temperature ranges with its Martensitic finish (M_f_) and Austenitic finish (A_f_) values as 30 °C and 60 °C, respectively. The temperature range inside the Stirling engine is also maintained at 40–60 °C. These temperature ranges match desired temperature values of the NiTiNOL spring to expand and contract, thus supplementing and enhancing the output power and efficiency of the Stirling engine.

Some parameters of NiTiNOL spring used in Stirnol engine are shown in Table 2.

After developing the Stirnol engine, its different properties were measured and compared to a Stirling engine of the same dimensions. To achieve this, a physical testing facility was developed and following properties were compared:

1. RMP (Revolutions per minute);
2. Brake Power;
3. Efficiency.

The results of experiments were encouraging, whereby the Stirnol engine displayed better results as compared to the Stirling engine. To further improve the research, the materials of both engines were changed to obtain optimum results. Initially, the lower plate (plate that conducts heat directly from the heat source) was selected as an aluminum material. Subsequently, the materials of the lower plates of both Stirling and Stirnol engines were changed and their properties were compared.

### 2.1. The Stirling Cycle vs. Stirnol Cycle

The thermodynamic power cycle concept is the basis for numerous thermal machines whose prime objective is to transform internal energy to mechanical work. The Stirling engine, being one of them, follows four closed regenerative cycles. The novel idea of Robert Stirling’s machine was a heat exchanger that absorbed the partial energy of heated gas exiting the expansion chamber to provide it when the cold gas returned to the chamber. Today, it is called a ’regenerator’, and in case of the LTD Stirling engine, this function is carried out by the displacer.

**Figure 4:**
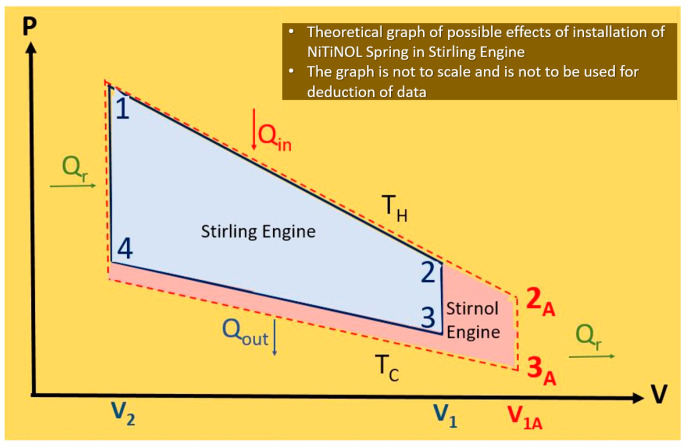
Theoretical graph depicting possible effects of installation of NiTiNOL spring in Stirling engine.

The heating substance or ideal gas experiences repeated expansions and compressions due to the heat exchanging from hot and cold temperatures. The step-wise theory of these processes of the Stirling engine and impact of the NiTiNOL spring in the Stirnol engine are described below. The same theory is graphically depicted in Figure 4.

#### 2.1.1. Isothermal Expansion/Spring Extraction

In the case of the Stirling engine, at steps 1–2, the gas attains heat from an external source and undertakes isothermal expansion. In the Stirnol engine, this isothermal expansion is augmented by the extractive motion of the NiTiNOL spring (as it expands on heating), theoretically capable of moving the process beyond points 2 to 2A.

#### 2.1.2. Isochoric Heat Removal

In the Stirling engine, at steps 2–3, the gas transfers heat to the displacer and follows an isochoric cooling process. The process is the same for the Stirnol engine.

#### 2.1.3. Isothermal Compression/Spring Retraction

In the Stirling engine, at steps 3–4, the gas undergoes an isothermal compression, transferring with the cool temperature source. For the Stirnol engine, there are three possibilities at this phase.

1. The shape memory alloy properties of the NiTiNOL spring cause it to retract to its original shape/size.
2. The downward movement of the displacer has to overcome the partial spring force that is already retracting due to a decrease in temperature. Some work would be wasted by the Stirnol engine in compressing the NiTiNOL spring.
3. If the NiTiNOL spring is attached to the displacer, it may have a pulling force on the piston, thereby augmenting the motion of the retraction stroke. This is not possible, due to the design limitations of the Stirnol engine.

#### 2.1.4. Isochoric Heat Addition

In steps 1–4, the compressed gas receives heat from the regenerator and follows an isochoric process.

### 2.2. Experimental Setup

**Figure 5:**
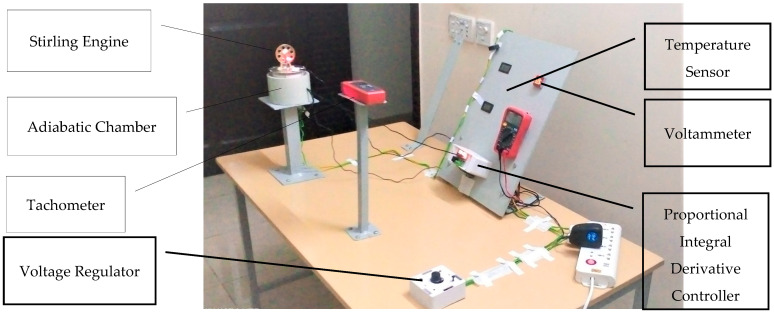
Testing facility for Experimentation.

To measure the differential parameters of the Stirling and Stirnol engine, an experimental setup was built (refer to Figure 5). The engine under observation was placed on top of an adiabatic cylinder-shaped chamber whose inner sides were sprayed with a lining of polyurethane material. The lining ensured minimal heat dissipation from the chamber to the atmosphere. A cut on top of the chamber ensured that the bottom plate of the engine was tightly inserted in it and heat was not dissipated from the top of the chamber. The heat was provided to the engine via a heating element placed above the polyurethane lining just below the lower plate of the engine.

#### 2.2.1. Torque Measurement

To measure the torque produced, the larger wheel of the engine was connected to a thread passing through a customized elevated pillar. In front of the engine was a digital tachometer fixed on a metallic pillar.

#### 2.2.2. RPM Measurement

RPM was measured by placing a tachometer in such a way that its laser beam hits the larger wheel of the engine.

#### 2.2.3. Gadgetry

The control panel consists of five gadgets:

1. Two digital thermometers;
2. Voltage/Ammeter;
3. Multimeter;
4. Proportional Integral Derivative (PID) Controller.

The digital thermometers have sensing probes attached to them. One probe is attached to the top plate of the engine that depicts T_c_ (temperature of cold side), while the other probe is attached to the lower plate of the engine and it reads T_h_ (temperature of hot side). The difference in both temperatures gives ∆T. The voltammeter shows the voltage and current that flow toward the heating element. The electric power to the heating element is routed through the PID controller that ensures that the desired temperature is maintained in the adiabatic chamber. A heat-sensing probe of the PID controller is placed in the adiabatic chamber and once the desired temperature inside the adiabatic chamber is sensed by the probe, it gives a signal to the PID controller that discontinues the input power to the heating element. Once the temperature inside the adiabatic chamber drops lower than the desired value, the probe signals the PID controller and the power is again provided to the heating element.

There is a Variac (voltage regulator) whose output gives power to the heating element. By changing the voltage through the Variac, the heat provided to the engine can be calculated from the formula Q = ∆Vi. The power provided to the heating element passes through two devices:

1. Variac (voltage regulator);
2. Proportional Integral Derivative (PID) controller.

**Figure 6:**
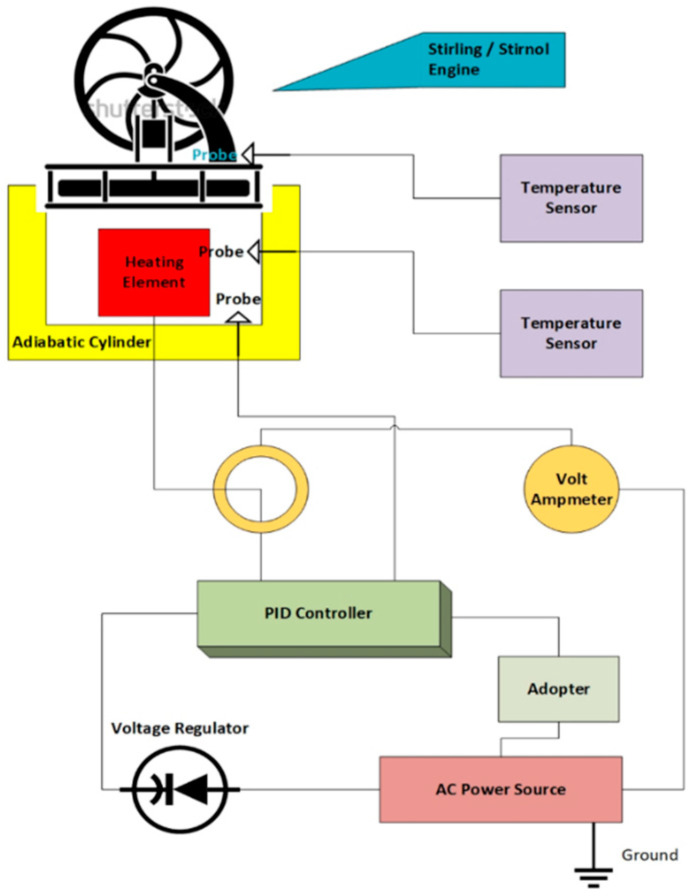
Wiring Diagram of Experimental setup.

The Variac changes the potential difference going to the heating element while the PID controller ensures the temperature inside the adiabatic chamber is kept constant. The wiring diagram of the experimental setup is shown in Figure 6.

## 3. Material Selection

Mother nature is full of unique materials and there are many more that have been artificially made by humans. Four types of material were selected as base plates for both Stirling and Stirnol engines and their properties are compared in the proceeding paragraphs. The most important criterion based on which the materials were selected was their thermal conductivities. In this regard, two materials were chosen with a high thermal conductivity: copper and aluminum, and two with lower thermal conductive values: brass and bronze. This was to have a comparison between the maxima and minima of the profile.

The plates are the intermediatory medium through which the heat is transferred from the adiabatic heating chamber to the Stirnol engine. The more conductive the medium, the more heat would be transferred and, subsequently, its RPM and efficiency would enhance. It is pertinent to mention that the Q_in_ to the adiabatic chamber for all material types is the same; however, due to changes in the conduction properties of plate material, the engine efficiency changes.

The properties of these materials are depicted in Table 3.

## 4. Analysis

The thermal efficiency of any engine is defined as the ratio of the power output to the heat transferred from high-temperature source. The first person to develop a model to predict the power output of a Stirling engine was Schmidt [13,14,15]. Subsequently, Kongtragool [16] proposed the modified Beale number with the following equation:

M Bn = [ẇ ÷ (pm Vp ƒ)] [ (1 + Ƭ) ÷ (1 − Ƭ)](1)

Typical M Bn values for LTD engines are 0.25 to 0.35. It is not practical to know the thermal efficiency and real power output from any thermal engine, but it can be calculated by obtaining measurements from primary variables. A testing facility was built to measure Stirnol and Stirling engine performances (refer to Figure 5). The testing procedure consists of supplying the heat QH (W) by electric means, and measuring voltage and electric current (QH = ΔVi). An increasing torque M is applied to the pulley on the flywheel shaft until the permanent state is achieved with constant shaft speed ω (where ẇ = M ω).

Three parameters were calculated and plotted, namely rotational speed, efficiency, and brake power.

### 4.1. Stirling and Stirnol Engines with Base Plate as Aluminum, Copper, Brass, and Bronze

Four different materials were chosen as base plates to conduct heat. The comparison of three parameters calculated and compared between Stirling and Stirnol engines for aluminum, copper, brass and bronze individually as the base plate is depicted in detail in the Supplementary Material.

The result of all materials is combined and described in the following paragraphs.

#### 4.1.1. Analysis of Temperature Difference vs. RPM

The performance comparison of both engines with different materials is shown in Table 4.

**Figure 7:**
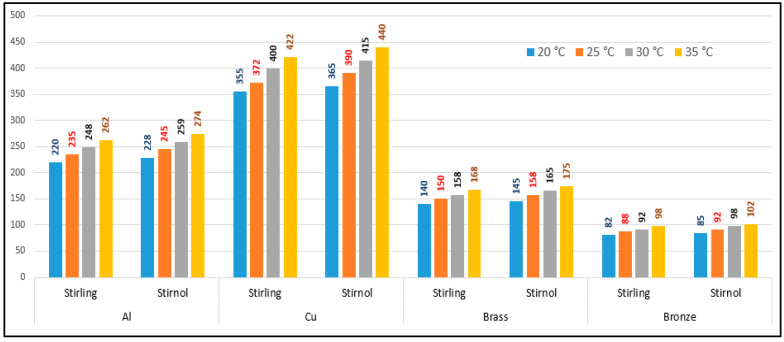
Performance Comparison of Stirling and Stirnol engines.

The performance comparison of both engines with different materials is shown in graphical form in Figure 7.

#### 4.1.2. Analysis of Brake Power vs. RPM

The brake power of an engine is the power available at the crankshaft. It is measured by the formula:

Brake Power = Torque × RPM(2)

Power (W) = T (N.m) × W (rad/sec)(3)

**Figure 8:**
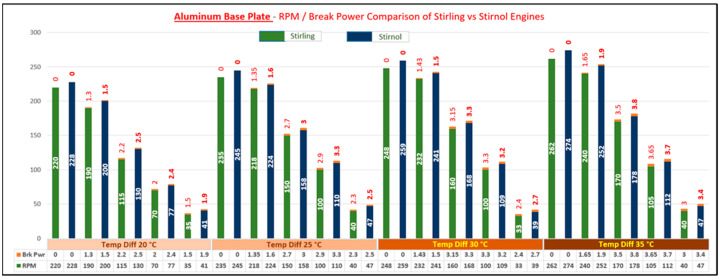
RPM vs. Brake Power Comparison of Stirling and Stirnol engines with the base plate as Aluminum.

**Figure 9:**
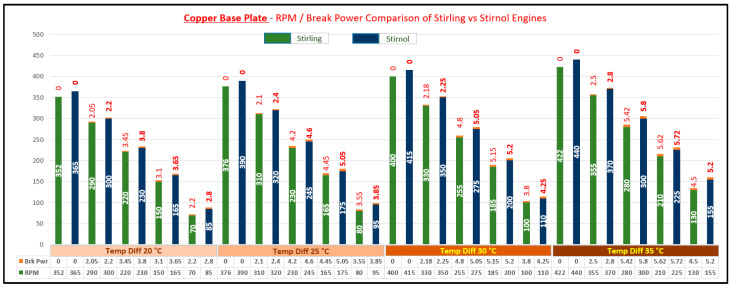
RPM vs. Brake Power Comparison of Stirling and Stirnol engines with the base plate as Copper.

**Figure 10:**
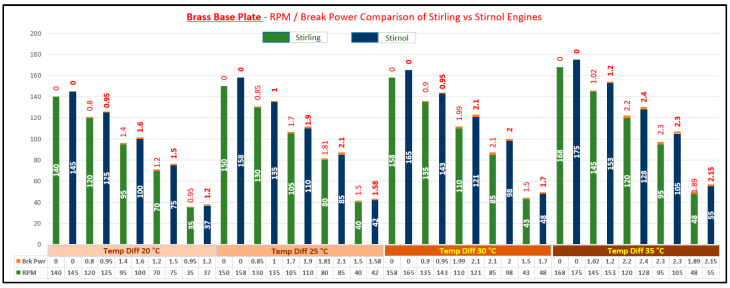
RPM vs. Brake Power Comparison of Stirling and Stirnol engines with the base plate as Brass.

**Figure 11:**
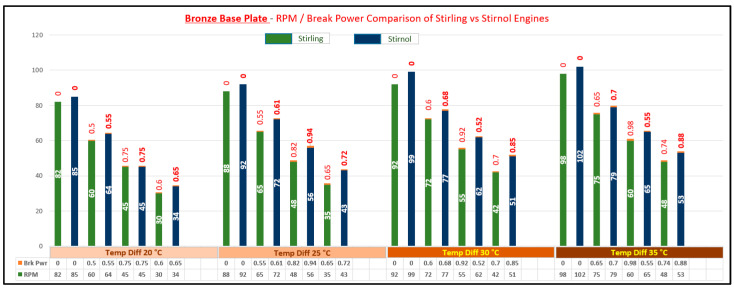
RPM vs. Brake Power Comparison of Stirling and Stirnol engines with the base plate as Bronze.

The result of said analysis is depicted in Table 5 and graphically shown in Figure 8, Figure 9, Figure 10 and Figure 11.

#### 4.1.3. Analysis of Engine Efficiency vs. RPM

Thermal Efficiency can be calculated by the following formula:

Efficiency = Useful Work done/Heat provided(4)

**Figure 12:**
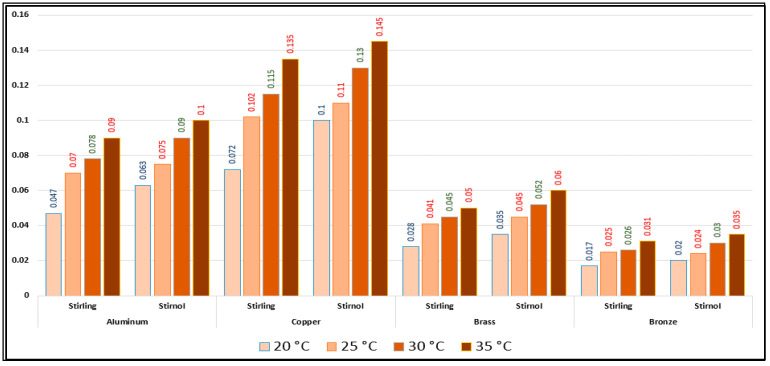
Comparative engine efficiencies with different materials of Stirling and Stirnol engines.

Optimum engine efficiencies for both engines with different materials are represented in Table 6 and Table 7 and graphically shown in Figure 12.

## 5. Results and Discussion

It can be rightly concluded that Low-Temperature-Differential (LTD) Stirling engines are simplest in design and cost-effective. The flipside of the argument is that these engines have very low efficiencies; however, they can utilize minimal waste heat energy from various sources. Senft [17] attested that the prospect to run an engine continuously with a temperature difference of only 0.5 °C and ΔT of about 30 °C is a good practical limit to produce mechanical power (e.g., using solar energy or waste heat from air conditioning systems). In this research, a Stirnol engine was introduced in which the NiTiNOL spring was merged into a Stirling engine. The comparison of Stirling and Stirnol engines revealed that the output efficiency of the Stirnol engine was slightly improved under the same input conditions. This research paper focused on analyzing the Stirnol engine while changing its base plate materials. The following results can be deduced from the experimental setup used to compute and compare the two engines with different materials:

(a) **Analysis of Temperature difference vs. RPM.** Both engines were subjected to similar temperatures by an even distribution of heat and subsequently changing materials of the lower plates to aluminum, copper, brass, and bronze. The temperature differences were kept constant by utilizing a PID controller whose purpose was to maintain a preset temperature. The RPM of flywheels of both engines was calculated by utilizing a laser-operated tachometer. The results obtained are displayed in Table 4 and graphically illustrated in Figure 7. It is depicted that the RPM of both the engines with the base plate as copper was enhanced owing to its better thermal conductivity as compared to the rest of its three compatriots. Bronze having the least thermal conductivity produced the slowest RPM values at the same temperature difference.
(b) **Analysis of Brake Power vs. RPM.** Brake power is the power developed by the engine at its output shaft. Traditionally, ‘brake power’ (bp) has been used as the definitive measurement of engine power. It is distinct from horsepower because it takes into account power loss due to friction. It is measured by running an engine up to full revolutions, and then letting it naturally slow down to a dead stop. The graphical representation of the brake power of both the engines with different materials is depicted in Table 5 and graphically shown in Figure 8, Figure 9, Figure 10 and Figure 11. The graph shows that the Stirnol engine with the base plate as copper showed a slightly different behavior below 250 RPM. Moreover, the graph of the Stirnol engine with the base plate as bronze also had some abrupt and unusual trend below 80 RPM, and the brake power of the Stirnol engine with the base plate as brass had a sluggish behavior below 110 RPM. It might be because of out-of-sync movement of the NiTiNOL spring with respect to the displacer.
(c) **Analysis of engine efficiency vs. RPM**. The engine efficiencies of both engines at different temperature differences are depicted in Table 6 and Table 7 for Stirling and Stirnol engines, respectively, and Figure 12 for both. The engines showed very low efficiencies as compared to the Carnot cycle; however, the positive side is that the presence of the NiTiNOL spring increased the efficiency in all cases with the change in material. The maximum efficiency of the Stirnol engine with the base plate as copper at 35 °C was calculated as 0.145%.

## 6. Conclusions

It can be concluded that the two domains of physics can be amalgamated to provide better and more useful results. The properties of SMAs can be better utilized and harnessed to improve the efficiency of the basic Stirling cycle at lower temperature differences. In this regard, an SMA (NiTiNOL spring) was incorporated into the Stirling engine. It was ensured that both engines were similar in geometry and arrangement, which were subjected to similar temperature differences. The experiment was carried out in a testing facility and the parameters calculated were RPM, efficiency, and brake power. The experiments were conducted by changing the materials of the base plates for both engines to aluminum, copper, brass, and bronze. The Stirnol engine showed slightly better performance than the Stirling engine, and with the application of enhanced thermal conductive materials, the efficiency was further improved.

The efficiency improvement with the incorporation of the NiTiNOL spring was lower; however, the experimentation was carried out on the very basic Low-Temperature Differential Stirling engine whose output efficiency was around 0.047–0.09% (aluminum as base plate). The research was conducted to share the novel idea of the effect of SMAs and different materials on the Stirling engine, which should open a window for researchers in the future who can get a lead and design more complex engines with better efficiencies. The crux of the research is that regardless of how meager the RPM increase is due to SMAs, there is still an improvement, and with better engine designs and improved Stirling engines to NiTiNOL combinations, a more efficient engine can be designed in the future.

## 7. Future Research Directions

Such an output can be used in many applications in the near future. By a systematic and rational sweeping of geometrical and physical parameters, one can compute the resulting heat transfer and fluid flows. A real machine improvement was identified and the present step shows the way for a more complete optimization. Indeed, the experiment will open new horizons for engineers in the future to venture into modifying the experimental setup, spring to displacer synchronization, and, more particularly, the choice of the SMA material and geometry.

## Figures and Tables

**Table 1:** Dimensions of Stirnol engine.

S No	Part	Dimensions
1	Diameter of the Upper and Lower plates	97 mm
2	The thickness of the Upper and Lower plates	1 mm
3	Diameter of Displacer	80 mm
4	Thickness of Displacer	10 mm
5	Height of Piston	15 mm
6	Diameter of Piston	12 mm
7	Diameter of Bigger Wheel	53 mm
8	Diameter of Smaller Wheel	15 mm
9	Diameter of Aluminum Cup	20 mm
10	Height of Aluminum Cup	5 mm
11	Diameter of NiTiNOL Spring	9 mm
12	Height of NiTiNOL Spring	5 mm

**Table 2:** NiTiNOL Spring characteristics.

Sr No	Parameters	Values
1	Spring Diameter	9 mm
2	Spring wire thickness	0.7 mm
3	Martensitic Finish Temperature	30 °C
4	Austenitic Finish Temperature	60 °C

**Table 3:** Material Characteristics.

S No	Metal	Density (kg/m^3^)	Melting Point (°C)	Thermal Conductivity (W/m °C)
1	Aluminum	2700	660	220
2	Copper	8900	1083	393.5
3	Brass	8450	950	130
4	Bronze	8730	1040	67

**Table 4:** Temperature difference vs. RPM Comparison of both engines.

S No	Temp of the Upper Plate	Temp of the Lower Plate	Temp Diff	Performance of Stirling Engine				Performance of Stirnol Engine			
				**Al**	**Cu**	**Brass**	**Bronze**	**Al**	**Cu**	**Brass**	**Bronze**
	**(°C)**			**RPM**				**RPM**			
1	25	45	20	220	355	140	82	228	365	145	85
2	25	50	25	235	372	150	88	245	390	158	92
3	25	55	30	248	400	158	92	259	415	165	98
4	25	60	35	262	422	168	98	274	440	175	102

**Table 5:** Analysis of Brake Power vs. RPM.

Ser	Temp Diff	Al Base Plate				Cu Base Plate				Brass Base Plate				Bronze Base Plate			
		Stirling		Stirnol		Stirling		Stirnol		Stirling		Stirnol		Stirling		Stirnol	
		RPM	Brk Pwr	RPM	Brk Pwr	RPM	Brk Pwr	RPM	Brk Pwr	RPM	Brk Pwr	RPM	Brk Pwr	RPM	Brk Pwr	RPM	Brk Pwr
1	20 °C	220	0	228	0	352	0	365	0	140	0	145	0	82	0	85	0
		190	1.3	200	1.5	290	2.05	300	2.2	120	0.8	125	0.95	60	0.5	64	0.55
		115	2.2	130	2.5	220	3.45	230	3.8	95	1.4	100	1.6	45	0.75	45	0.75
		70	2	77	2.4	150	3.1	165	3.65	70	1.2	75	1.5	30	0.6	34	0.65
		35	1.5	41	1.9	70	2.2	85	2.8	35	0.95	37	1.2				
2	25 °C	235	0	245	0	376	0	390	0	150	0	158	0	88	0	92	0
		218	1.35	224	1.6	310	2.1	320	2.4	130	0.85	135	1	65	0.55	72	0.61
		150	2.7	158	3	230	4.2	245	4.6	105	1.7	110	1.9	48	0.82	56	0.94
		100	2.9	110	3.3	165	4.45	175	5.05	80	1.81	85	2.1	35	0.65	43	0.72
		40	2.3	47	2.5	80	3.55	95	3.85	40	1.5	42	1.58				
3	30 °C	248	0	259	0	400	0	415	0	158	0	165	0	92	0	99	0
		232	1.43	241	1.5	330	2.18	350	2.25	135	0.9	143	0.95	72	0.6	77	0.68
		160	3.15	168	3.3	255	4.8	275	5.05	110	1.99	121	2.1	55	0.92	62	0.52
		100	3.3	109	3.2	185	5.15	200	5.2	85	2.1	98	2	42	0.7	51	0.85
		33	2.4	39	2.7	100	3.8	110	4.25	43	1.5	48	1.7				
4	35 °C	262	0	274	0	422	0	440	0	168	0	175	0	98	0	102	0
		240	1.65	252	1.9	355	2.5	370	2.8	145	1.02	153	1.2	75	0.65	79	0.7
		170	3.5	178	3.8	280	5.42	300	5.8	120	2.2	128	2.4	60	0.98	65	0.55
		105	3.65	112	3.7	210	5.62	225	5.72	95	2.3	105	2.3	48	0.74	53	0.88
		40	3	47	3.4	130	4.5	155	5.2	48	1.89	55	2.15				

**Table 6:** Engine efficiency vs. RPM of Stirling engine.

S No	Temp Diff (°C)	Aluminum	Copper	Brass	Bronze
		Max Efficiency (%)	Max Efficiency (%)	Max Efficiency (%)	Max Efficiency (%)
1	20	0.047	0.072	0.028	0.017
2	25	0.07	0.102	0.041	0.025
3	30	0.078	0.115	0.045	0.026
4	35	0.09	0.135	0.05	0.031

**Table 7:** Engine efficiency vs. RPM of Stirnol engine.

S No	Temp Diff (°C)	Aluminum	Copper	Brass	Bronze
		Max Efficiency (%)	Max Efficiency (%)	Max Efficiency (%)	Max Efficiency (%)
1	20	0.063	0.1	0.035	0.02
2	25	0.075	0.11	0.045	0.024
3	30	0.09	0.13	0.052	0.03
4	35	0.1	0.145	0.06	0.035

## Data Availability

The datasets analyzed during the current study are available from the corresponding author on reasonable request.

## Supplementary Materials

The following supporting information can be downloaded at: https://www.mdpi.com/article/10.3390/ma16083257/s1, Four different materials were chosen as a base plate to conduct heat. The comparison of three parameters calculated and compared between Stirling and Stirnol engines for aluminum, copper, brass and bronze individually as the base plate is depicted in detail in the supplementary material.
