# Supplementary material for: Effect of Material Change on Stirnol Engine: A Combination of NiTiNOL (Shape Memory Alloy) and Gamma Stirling Engine
Source: Materials (Basel). 2023 Apr 20;16(8):3257. doi: 10.3390/ma16083257 (PMC10146608; doi:10.3390/ma16083257)
Supplement: Supplementary file 1 [file materials-16-03257-s001.zip › materials-2191710-supplementary.pdf]

materials

MDPI

Supplementary Material

Effect of Material Change on Stirnol Engine: A Combination of NiTiNOL (Shape Memory Alloy) and Gamma Stirling Engine

Humayun Arif [1,*], Aqueel Shah [1], Tahir Abdul Hussain Ratlamwala [1], Khurram Kamal [1] and Maqsood Ahmed Khan [2]

[1] Department of I&ME, PNEC Pakistan Navy Engineering College, NUST, Karachi 75350, Pakistan; aqueel@pnec.nust.edu.pk (A.S.); tahir.ratlamwala@pnec.nust.edu.pk (T.A.H.R.); khurram.kamal@pnec.nust.edu.pk (K.K.)
[2] Department of I&ME, NED University of Engineering and Technology, Karachi 75350, Pakistan; maqsoodahmed@neduet.edu.pk
* Correspondence: humayun1511@yahoo.com; Tel.: +92-332-5137091

Citation: Arif, H.; Shah, A.; Ratlamwala, T.A.H.; Kamal, K.; Khan, M.A. Effect of Material Change on Stirnol Engine: A Combination of NiTiNOL (Shape Memory Alloy) and Gamma Stirling Engine. *Materials* **2023**, *16*, 3257. https://doi.org/10.3390/ma16083257

Academic Editor: Zongbin Li

Received: 14 January 2023
Revised: 7 March 2023
Accepted: 10 March 2023
Published: 20 April 2023

Copyright: © 2023 by the authors. Licensee MDPI, Basel, Switzerland. This article is an open access article distributed under the terms and conditions of the Creative Commons Attribution (CC BY) license (https:// creativecommons.org/licenses/by/ 4.0/).

S1. Stirling and Stirnol engines with base plate as aluminum

The different parameters calculated experimentally and further compared between a Stirling engine and Stirnol engine having base plates as aluminum is described in following paragraphs.

S1.1. Analysis of Temperature difference vs RPM

Table S1. Temperature difference vs RPM comparison of both engines.

S No.	Temp of upper plate	Temp of lower plate	Temp diff	Performance of Stirling engine	Performance of Stirnol engine
	(°C)	(°C)	(°C)	RPM	RPM
1	25	45	20	220	228
2	25	50	25	235	245
3	25	55	30	248	259
4	25	60	35	262	274

The Performance comparison of both the engines is shown in graphical form in Figure S1.

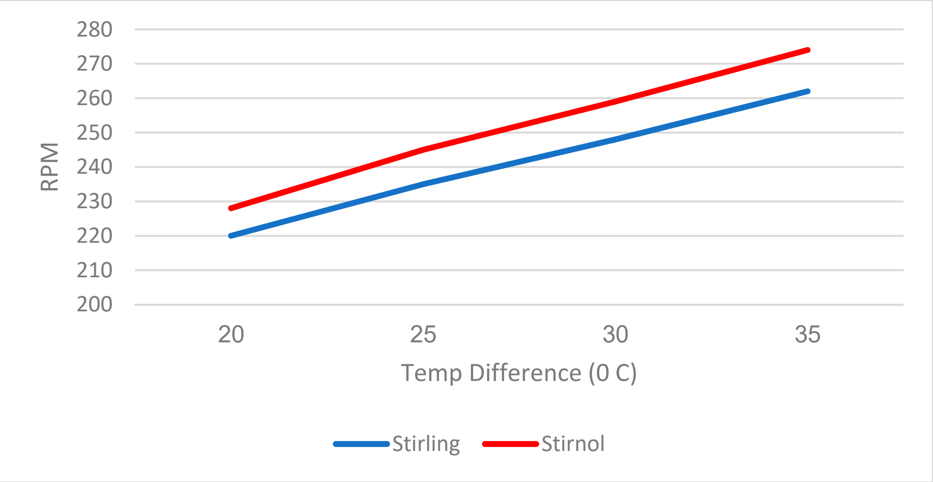

Figure S1. Performance comparison of Stirling and Stirnol engines.

S1.2. Analysis of Brake power vs RPM

The brake power of an IC engine is the power available at the crankshaft. It is measured by the formula:

$$\text{Brake power} = \text{Torque} * \text{RPM} \quad (S1)$$

$$\text{Power (W)} = \text{T (N.m)} * \text{W (rad/sec)} \quad (S2)$$

Table S2. Analysis of Brake power vs RPM.

S No.	Temp diff (°C)	Stirling engine RPM	Stirling engine Brake power (mW)	Stirnol engine RPM	Stirnol engine Brake power (mW)
1	20	220	0	228	0
		190	1.3	200	1.5
		115	2.2	130	2.5
		70	2.0	77	2.4
		35	1.5	41	1.9
2	25	235	0	245	0
		218	1.35	224	1.6
		150	2.7	158	3.0
		100	2.9	110	3.3
		40	2.3	47	2.5
3	30	248	0	259	0
		232	1.43	241	1.5
		160	3.15	168	3.3
		100	3.3	109	3.2
		33	2.4	39	2.7
4	35	262	0	274	0
		240	1.65	252	1.9
		170	3.5	178	3.8
		105	3.65	112	3.7
		40	3.0	47	3.4

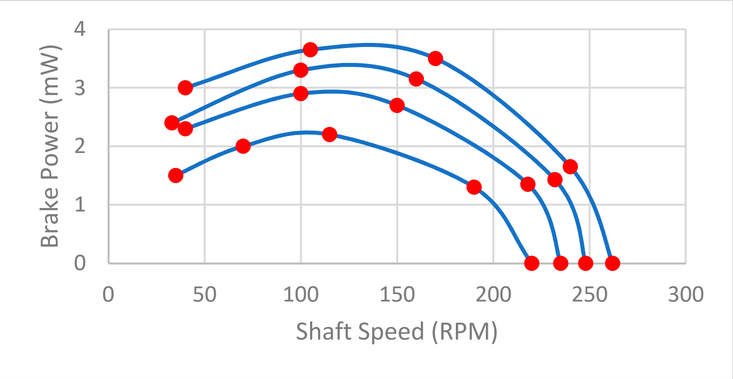

Figure S2. Brake power vs shaft speed of Stirling engine.

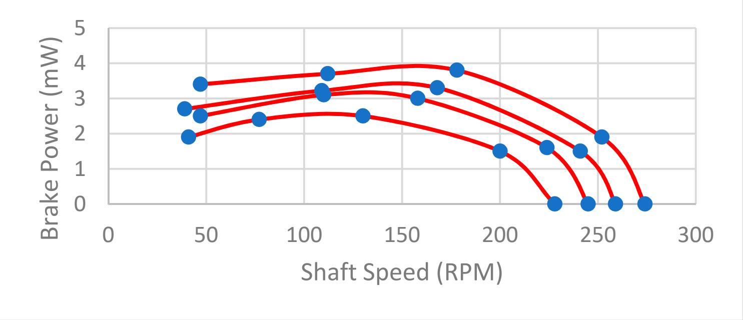

Figure S3. Brake power vs shaft speed of Stirnol engine.

S1.3. Analysis of Engine efficiency vs RPM

Thermal efficiency can be calculated by the following formula:

$$\text{Efficiency} = \text{Useful work done} / \text{Heat provided} \quad (S3)$$

Table S3. Engine efficiency vs RPM of Stirling engine.

S No	Temp diff (°C)	Aluminum - Max efficiency (%)
1	20	0.047
2	25	0.07
3	30	0.078
4	35	0.09

Table S4. Engine efficiency vs RPM of Stirnol engine.

S No	Temp diff (°C)	Aluminum - Max efficiency (%)
1	20	0.063
2	25	0.075
3	30	0.09
4	35	0.1

S2. Stirling and Stirnol engines with base plate as copper

The second material used to conduct heat was selected as copper. The different parameters calculated experimentally and further compared between a Stirling engine and Stirnol engine having base plates as copper is described in following paragraphs.

S2.1. Analysis of Temperature difference vs RPM

Table S5. Temp difference vs RPM comparison of both engines.

S No.	Temp of upper plate	Temp of lower plate	Temp diff	Performance of Stirling engine	Performance of Stirnol engine
	(°C)	(°C)	(°C)	RPM	RPM
1	25	45	20	355	365
2	25	50	25	372	390
3	25	55	30	400	415
4	25	60	35	422	440

The Performance comparison of both the engines is shown in graphical form in Figure S4.

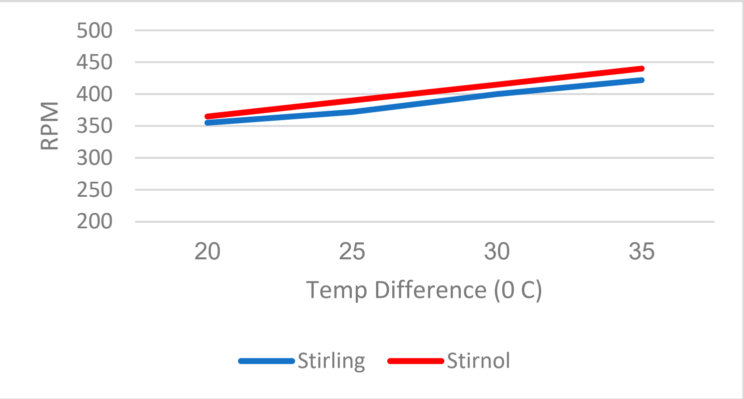

Figure S4. Performance comparison of Stirling and Stirnol engines.

S2.2. Analysis of Brake power vs RPM

The brake power of an IC engine is the power available at the crankshaft. It is measured by the formula:

$$\text{Brake power} = \text{Torque} * \text{RPM} \quad (S4)$$

$$\text{Power (W)} = \text{T (N.m)} * \text{W (rad/sec)} \quad (S5)$$

Table S6. Analysis of Brake power vs RPM.

S No.	Temp diff (°C)	Stirling engine		Stirnol engine	
		RPM	Brake power (mW)	RPM	Brake power (mW)
1	20	352	0	365	0
		290	2.05	300	2.2
		220	3.45	230	3.8
		150	3.1	165	3.65
		70	2.2	85	2.8
2	25	376	0	390	0
		310	2.1	320	2.4
		230	4.2	245	4.6
		165	4.45	175	5.05
		80	3.55	95	3.85
3	30	400	0	415	0
		330	2.18	350	2.25
		255	4.8	275	5.05
		185	5.15	200	5.2
		100	3.8	110	4.25
4	35	422	0	440	0
		355	2.5	370	2.8
		280	5.42	300	5.8
		210	5.62	225	5.72
		130	4.5	155	5.2

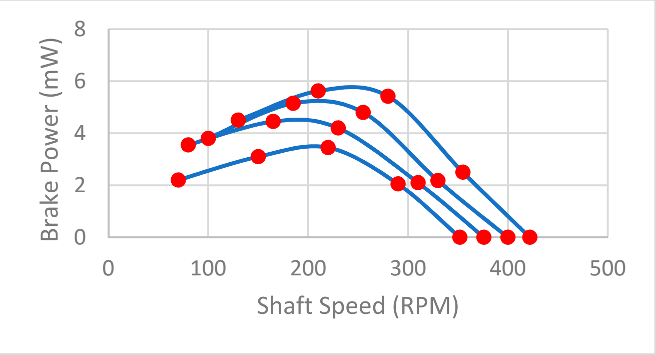

Figure S5. Brake power vs shaft speed of Stirling engine.

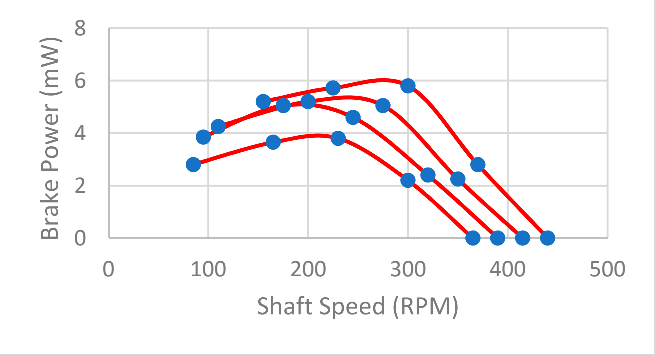

Figure S6. Brake power vs shaft speed of Stirnol engine.

S2.3. Analysis of Engine efficiency vs RPM

Thermal efficiency can be calculated by the following formula:

Efficiency = Useful work done / Heat provided (S6)

Table S7. Engine efficiency vs RPM of Stirling engine.

S No	Temp diff (°C)	Copper - Max efficiency (%)
1	20	0.072
2	25	0.102
3	30	0.115
4	35	0.135

Table S8. Engine efficiency vs RPM of Stirnol engine.

S No	Temp diff (°C)	Copper - Max efficiency (%)
1	20	0.1
2	25	0.11
3	30	0.13
4	35	0.145

S3. Stirling and Stirnol engines with base plate as brass

The fourth material used to conduct heat was selected as brass. The different parameters calculated experimentally and further compared between a Stirling engine and Stirnol engine having base plates as brass is described in following paragraphs.

S3.1. Analysis of Temperature difference vs RPM

Table S9. Temp difference vs RPM comparison of both engines.

S No.	Temp of upper plate (°C)	Temp of lower plate (°C)	Temp diff (°C)	Performance of Stirling engine RPM	Performance of Stirnol engine RPM
1	25	45	20	140	145
2	25	50	25	150	158
3	25	55	30	158	165
4	25	60	35	168	175

The Performance comparison of both the engines is shown in graphical form in Figure S7.

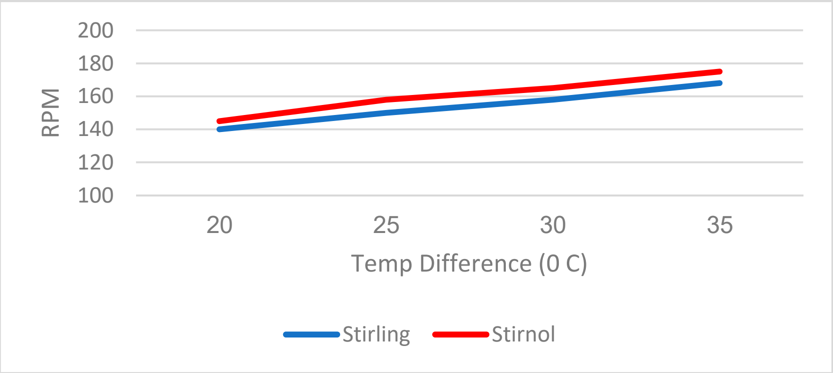

Figure S7. Performance comparison of Stirling and Stirnol engines.

S3.2. Analysis of Brake power vs RPM

The brake power of an IC engine is the power available at the crankshaft. It is measured by the formula:

$$\text{Brake power} = \text{Torque} * \text{RPM} \quad (S7)$$

$$\text{Power (W)} = \text{T (N.m)} * \text{W (rad/sec)} \quad (S8)$$

Table S10. Analysis of Brake power vs RPM.

S No.	Temp diff (°C)	Stirling engine		Stirnol engine	
		**RPM**	**Brake power (mW)**	**RPM**	**Brake power (mW)**
1	20	140	0	145	0
		120	0.8	125	0.95
		95	1.4	100	1.6
		70	1.2	75	1.5
		35	0.95	37	1.2
2	25	150	0	158	0
		130	0.85	135	1.0
		105	1.7	110	1.9
		80	1.81	85	2.1
		40	1.5	42	1.58
3	30	158	0	165	0
		135	0.9	143	0.95
		110	1.99	121	2.1
		85	2.1	98	2.0
		43	1.5	48	1.7
4	35	168	0	175	0
		145	1.02	153	1.2
		120	2.2	128	2.4
		95	2.3	105	2.3
		48	1.89	55	2.15

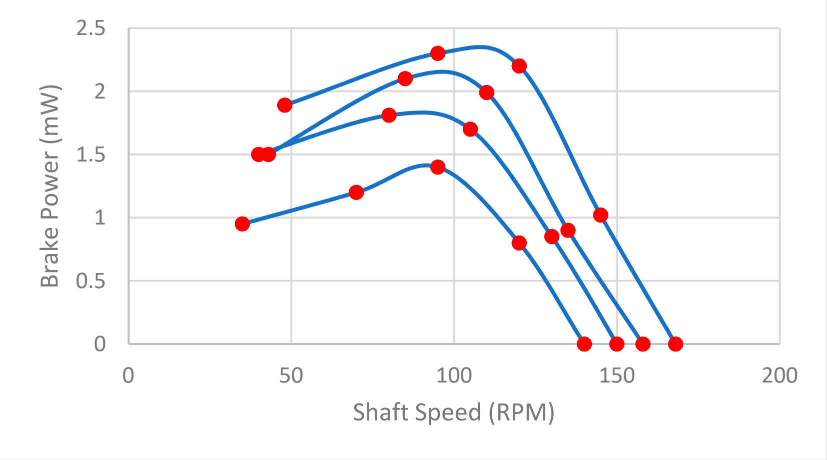

Figure S8. Brake power vs shaft speed of Stirling engine.

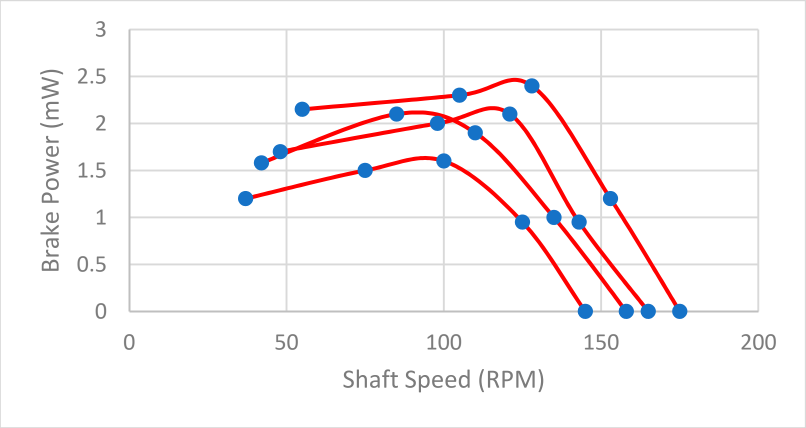

Figure S9. Brake power vs shaft speed of Stirnol engine.

S3.3 Analysis of Engine efficiency vs RPM

Thermal efficiency can be calculated by the following formula:

$$\text{Efficiency} = \text{Useful work done} / \text{Heat provided} \tag{S9}$$

Table S11. Engine efficiency vs RPM of Stirling engine.

S No	Temp diff (°C)	Brass - Max efficiency (%)
1	20	0.028
2	25	0.041
3	30	0.045
4	35	0.05

Table S12. Engine efficiency vs RPM of Stirnol engine.

S No	Temp diff (°C)	Brass - Max efficiency (%)
1	20	0.035
2	25	0.045
3	30	0.052
4	35	0.06

S4. Stirling and Stirnol engines with base plate as bronze

The third material used to conduct heat was selected as bronze. The different parameters calculated experimentally and further compared between a Stirling engine and Stirnol engine having base plates as bronze is described in following paragraphs.

S4.1. Analysis of Temperature difference vs RPM

Table S13. Temp difference vs RPM comparison of both engines.

S No.	Temp of upper plate	Temp of lower plate	Temp diff	Performance of Stirling engine	Performance of Stirnol engine
	(°C)	(°C)	(°C)	RPM	RPM
1	25	45	20	82	85
2	25	50	25	88	92
3	25	55	30	92	98
4	25	60	35	98	102

The Performance comparison of both the engines is shown in graphical form in Figure S10 which depicts that SMA has augmented the overall efficiency of the engine.

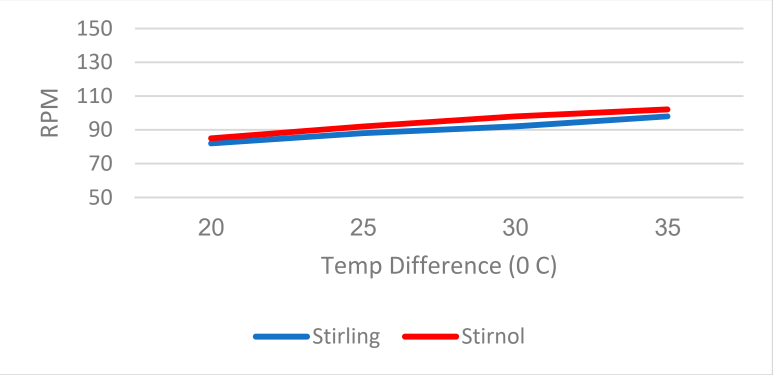

Figure S10. Performance comparison of Stirling and Stirnol engines.

S4.2. Analysis of Brake power vs RPM

The brake power of an IC engine is the power available at the crankshaft. It is measured by the formula:

$$\text{Brake power} = \text{Torque} * \text{RPM} \quad \text{(S10)}$$

$$\text{Power (W)} = \text{T (N.m)} * \text{W (rad/sec)} \quad \text{(S11)}$$

Table S14. Analysis of Brake power vs RPM.

S No.	Temp diff (°C)	Stirling engine		Stirnol engine	
		RPM	Brake power (mW)	RPM	Brake power (mW)
1	20	82	0	85	0
		60	0.5	64	0.55
		45	0.75	45	0.75
		30	0.6	34	0.65
2	25	88	0	92	0
		65	0.55	72	0.61
		48	0.82	56	0.94
		35	0.65	43	0.72
3	30	92	0	99	0
		72	0.6	77	0.68
		55	0.92	62	0.52
		42	0.7	51	0.85
4	35	98	0	102	0
		75	0.65	79	0.70
		60	0.98	65	0.55
		48	0.74	53	0.88

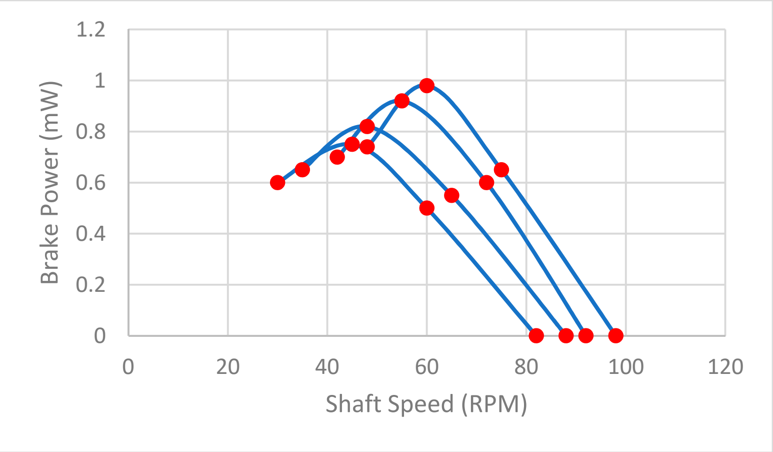

Figure S11. Brake power vs shaft speed of Stirling engine.

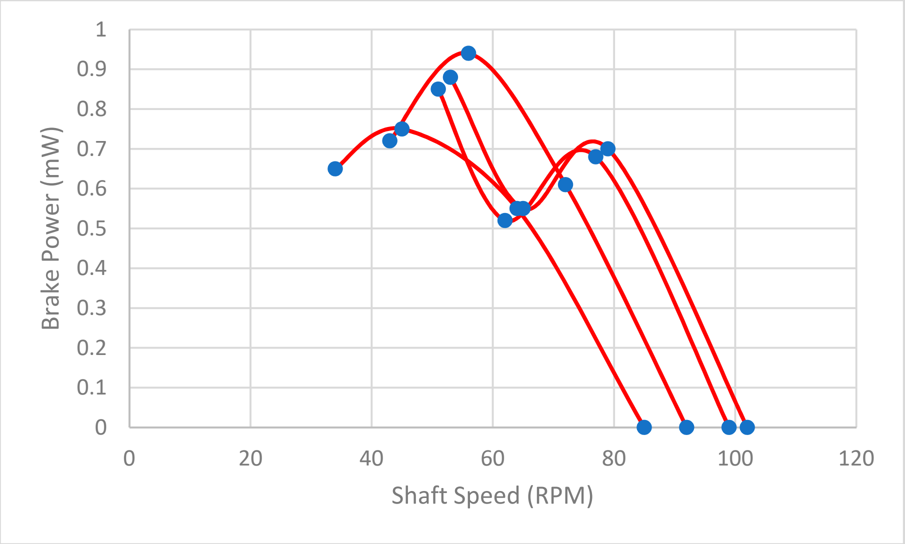

Figure S12. Brake power vs shaft speed of Stirnol engine.

S4.3. Analysis of Engine efficiency vs RPM

Thermal efficiency can be calculated by the following formula:

$$\text{Efficiency} = \text{Useful work done} / \text{Heat provided} \quad (S12)$$

Table S15. Engine efficiency vs RPM of Stirling engine.

S No	Temp diff (°C)	Bronze - Max efficiency (%)
1	20	0.017
2	25	0.025
3	30	0.026
4	35	0.031

Table S16. Engine efficiency vs RPM of Stirnol engine.

S No	Temp diff (°C)	Bronze - Max efficiency (%)
1	20	0.02
2	25	0.024
3	30	0.03
4	35	0.035
